# A behavioral task with more opportunities for memory acquisition promotes the survival of new neurons in the adult dentate gyrus

**DOI:** 10.1038/s41598-018-25331-w

**Published:** 2018-05-09

**Authors:** Ida E. J. Aasebø, Ameya Sanjay Kasture, Marzia Passeggeri, Ayumu Tashiro

**Affiliations:** 1 Kavli Institute for Systems Neuroscience, Norwegian University of Science and Technology, Trondheim, Norway; 2 Warwick-NTU Neuroscience Research Programme, School of Biological Sciences, Nanyang Technological University, Singapore and School of Life Sciences, University of Warwick, Coventry, UK; 3 School of Biological Sciences, Nanyang Technological University, Singapore, Singapore

## Abstract

It has been suggested that the dentate gyrus, particularly its new neurons generated via adult neurogenesis, is involved in memory acquisition and recall. Here, we trained rats in two types of Morris water maze tasks that are differentially associated with these two memory processes, and examined whether new neurons are differently affected by the two tasks performed during the second week of neuronal birth. Our results indicate that the task involving more opportunities to acquire new information better supports the survival of new neurons. Further, we assessed whether the two tasks differentially induce the expression of an immediate early gene, Zif268, which is known to be induced by neuronal activation. While the two tasks differentially induce Zif268 expression in the dentate gyrus, the proportions of new neurons activated were similar between the two tasks. Thus, we conclude that while the two tasks differentially activate the dentate gyrus, the task involving more opportunities for memory acquisition during the second week of the birth of new neurons better promotes the survival of the new neurons.

## Introduction

The mammalian brain continues to generate new neurons in a few select regions throughout life^1^. One of those regions is the dentate gyrus, a subregion of the hippocampus that is crucial in cognitive functions such as learning and memory^2^. Neural stem/progenitor cells located in the dentate gyrus generate granule cells, which are the principal neuronal type in the dentate gyrus. Newly born neurons undergo a long course of developmental processes and functionally integrate into existing neuronal circuits. Accumulating evidence has suggested that these new neurons play a role in learning and memory^3–7^.

Memory involves different subprocesses, such as the processes of acquisition and recall. Some behavioral and computational studies have particularly emphasized the role of the dentate gyrus in memory acquisition^2,8–12^, while other behavioral studies have supported the role of the dentate gyrus in memory recall^13–16^. Because these two memory subprocesses have different computational demands, we reason that acquisition and recall would rely on the dentate gyrus in dissimilar manners and would have different effects on new neurons.

On the basis of this reasoning, we decided to examine whether behavioral tasks that are associated with memory acquisition and recall to different degrees affect new neurons differentially. Previous studies have shown that the properties of new neurons, including their survival and activation, are affected by various factors, such as learning-related experience, specifically in the second week of neuronal birth^17–19^. Therefore, we tested how the survival and activation of new neurons are affected by training for two memory tasks that are differentially associated with memory acquisition and recall, performed during this maturational period.

For this purpose, we trained rats in two different versions of the water maze task: a reference memory task and a working memory task. In the reference memory task, rats were trained to find a platform that was located in a fixed position over the whole training period^20^. Early in the training period, rats would need to acquire various pieces of information relevant to solving the task, e.g., 1) the existence of the hidden platform, 2) the fact that climbing onto the platform leads to escape from the water, and 3) the location of the platform. Later in the training period, rats would recall the previously acquired information regarding the platform position and strengthen the acquired information. In the working memory task, the platform was moved to a new position every day^21^. As in the reference memory task, rats would need to acquire information on solving the task early in the training period. However, in contrast to the reference memory task, rats would need to acquire new information every day regarding the platform location, such as 1) the fact that the platform is no longer located in the original position, 2) the fact that the platform is located in a new position, and 3) the new location of the platform. Therefore, in the working memory task, rats are confronted with more opportunities to acquire new information than in the reference memory task, at the expense of opportunities to recall previously acquired information.

## Results

### Performance in Morris water maze tasks

**Figure 1:**
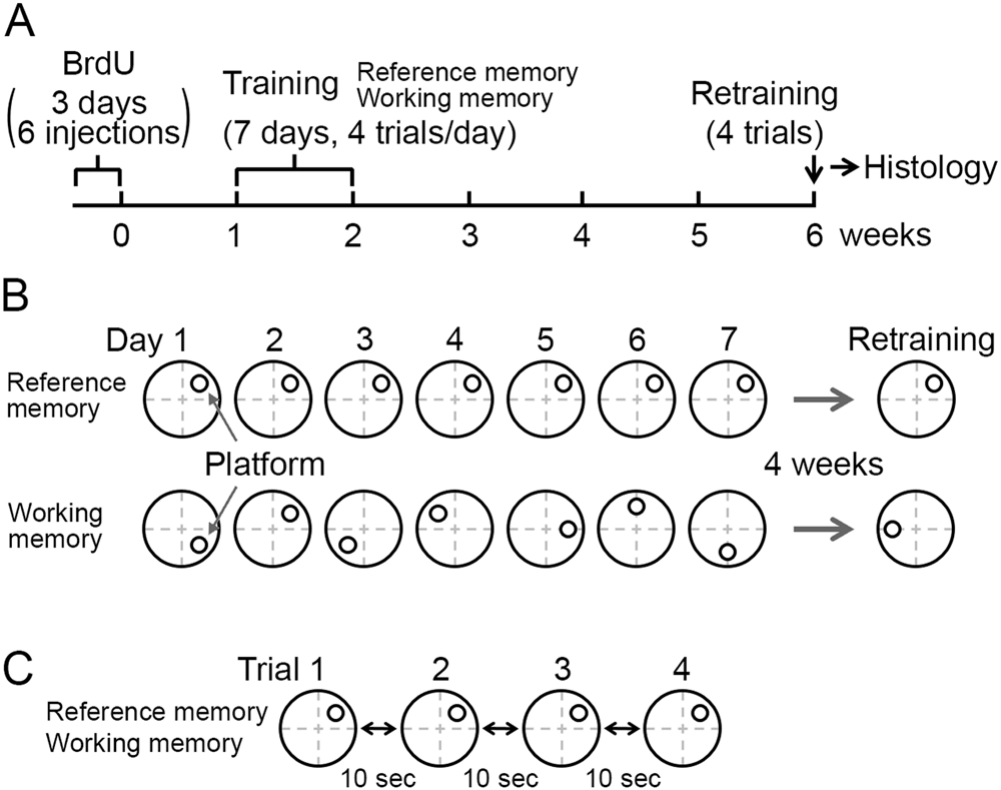
Experimental design. (**A**) Experimental timeline. (**B**) Reference memory and working memory versions of the Morris water maze task. The platform location was kept constant across all training days for the reference memory group, whereas the location was changed every day for the working memory group. (**C**) Within training days, the platform location was kept constant.

The experimental timeline is described in Fig. 1 (see Methods for details). One week after the BrdU injections, we began training two groups of rats in either the reference memory or the working memory task, with four trials per day for 7 consecutive days. For the reference memory group (n = 7 rats), the platform location remained unchanged, whereas, for the working memory group (n = 8 rats), the platform was moved to a new position every day. Six weeks after the BrdU injections, rats were retrained on the same tasks for four trials.

**Figure 2:**
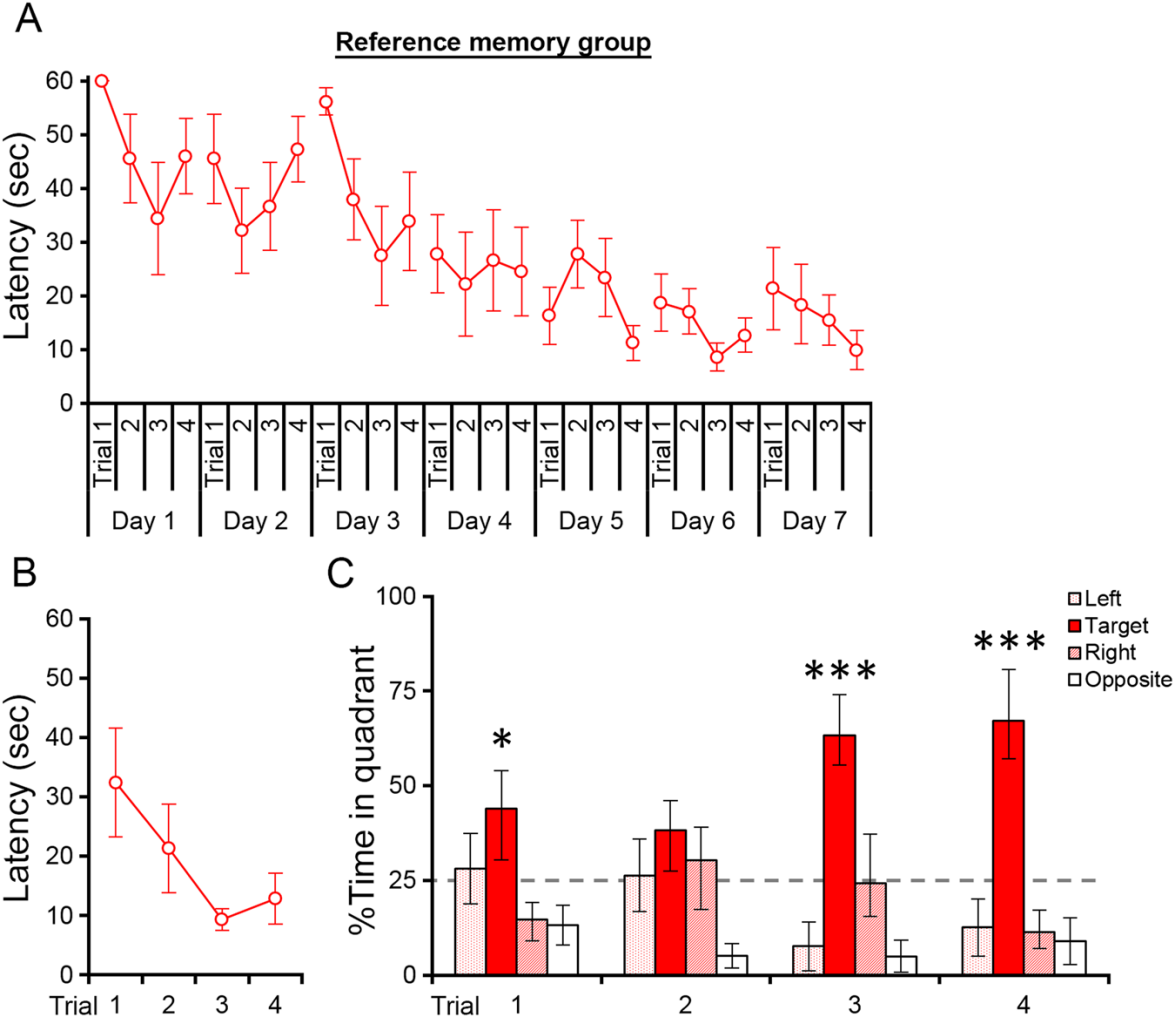
Performance of the reference memory group in the water maze task. (**A**) Latencies to reach the platform in individual trials during days 1–7 of the training period. (**B**) Latencies to reach the platform in individual trials on the retraining day. (**C**) Percentages of time spent in each quadrant during trials 1–4 on the retraining day. The platform was in the target quadrant. *p < 0.05, ***p < 0.005 in a one-way ANOVA. All data are shown as the mean ± s.e.m.

The latencies to reach the platform for individual trials of the reference memory group are shown in Fig. 2A. As expected from similar reference memory water maze tasks in previous studies in rats^22,23^, latencies decreased over the first 4 days and then approached an asymptotic level of performance. We performed two-way repeated measures ANOVA (Day × Trial) for latencies over 7 days of the training period (days 1–7). A significant effect of Day was found (p < 1 × 10^−5^). Post hoc pairwise comparisons showed significant reductions on days 4, 5, 6, and 7 compared with day 1 (p < 0.05 in the least significant difference [LSD] test) but no significant difference among days 4, 5, 6, and 7 (p > 0.05 in the LSD test). The effects of Trial and Day × Trial interaction were not significant (p > 0.05). Thus, the latencies to reach the platform indicate that rats in the reference memory group learned the fixed platform position over the 7 days of training.

Four retraining trials were performed 4 weeks after the last training day (Fig. 2B). To verify that rats in the reference memory group retained the acquired memory of the platform position on the retraining day, we performed two analyses. First, we compared the trial 1 latencies between day 1 and the retraining day. We detected the latency on the retraining day to be significantly lower than that on day 1 (p < 0.03 in a two-tailed paired *t*-test). Second, we calculated the percentages of time that the rats spent in each of the four quadrants of the water maze on the retraining day (Fig. 2C). In trial 1 of the retraining day, we found that the rats showed a significant bias in time spent among the four quadrants (p < 0.04 in one-way ANOVA). Specifically, the rats spent the highest percentage of time in the target quadrant that contained the platform (44.0 ± 9.99%, mean ± s.e.m.). The results of these two analyses support the interpretation that rats in the reference memory group remembered the learned platform position at the beginning of the retraining day.

**Figure 3:**
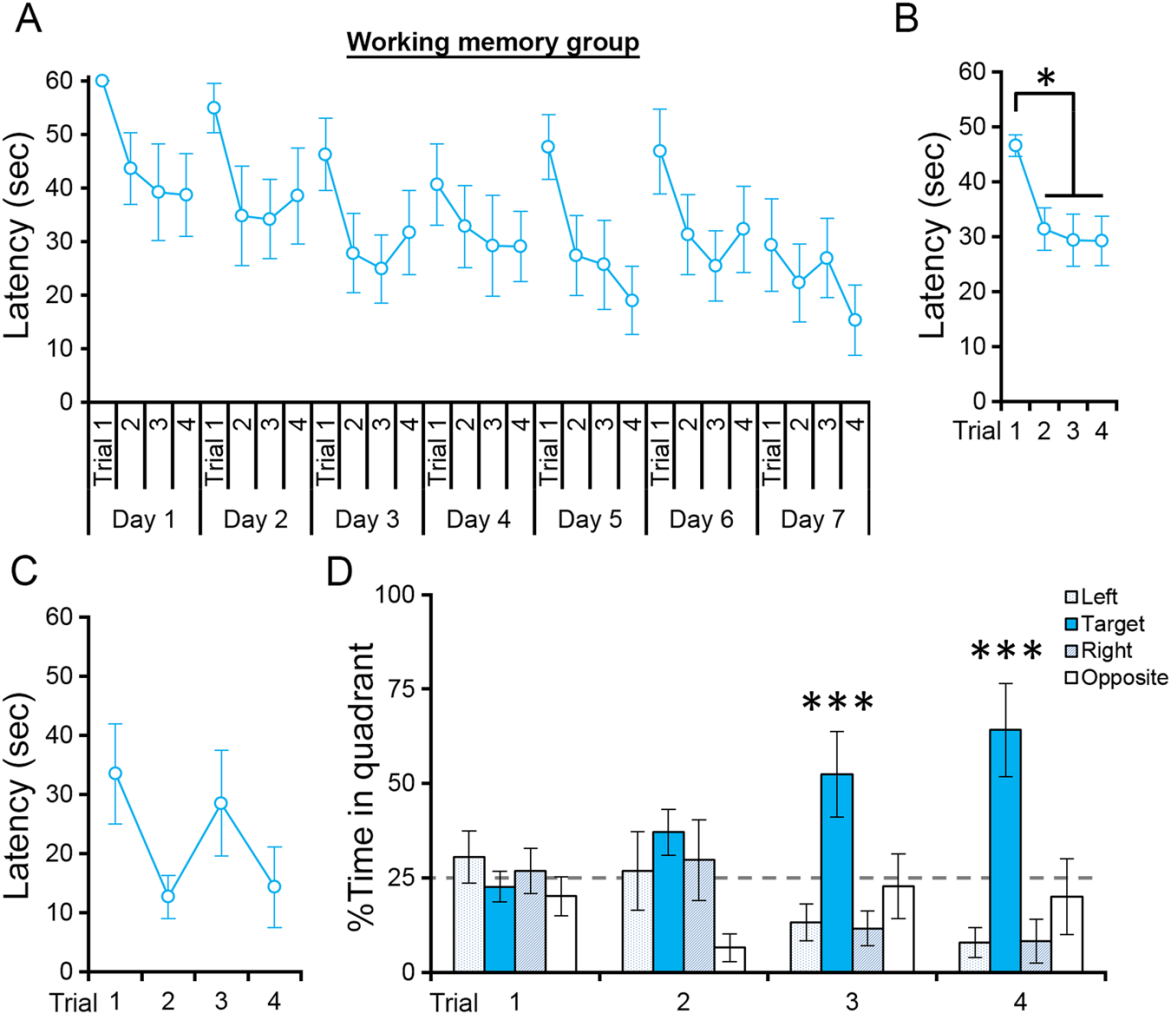
Performance of the working memory group in the water maze task. (**A**) Latencies to reach the platform in individual trials during days 1–7 of the training period. (**B**) Latencies to reach the platform in trials 1–4 averaged over the 7-day training period. *p < 0.05 in post hoc LSD test. (**C**) Latencies to reach the platform in individual trials on the retraining day. (**D**) Percentages of time spent in each quadrant in trials 1–4 on the retraining day. The platform was in the target quadrant. ***p < 0.005 in a one-way ANOVA. All data are shown as the mean ± s.e.m.

Figure 3A shows the latencies to reach the platform for individual trials in the working memory group. Although there were some variations among days, we found large reductions in latency to reach the platform from the first to the second trials of the days, as expected from comparable working memory water maze tasks in a previous study with rats^21^. We performed a two-way repeated measures ANOVA (Day × Trial) for latencies over the 7 days and detected significant effects of Day (p < 0.01) and Trial (p < 0.001) but not of Day × Trial interaction (p = 0.982). Significant reductions were detected from day 1 to days 4, 5, and 7 (p < 0.05 in post hoc LSD test) and from trial 1 to trials 2, 3, and 4 (p < 0.01, in post hoc LSD test). The differences among trials 2, 3, and 4 were not significant (p > 0.5). Figure 3B shows latencies in the four trials averaged over the 7 days of the training period. On average, latencies were reduced by 15.1 s from the first to the second trial and by only 2.2 s from the second to the fourth trial. These results are consistent with a previous study in that rats showed larger reductions in latency from the first to the second trial of the day than between any other consecutive trials^21^, suggesting that rats in the working memory group learned the new platform positions in the first trial.

To verify that rats in the working memory group learned new platform positions on the retraining day, we performed two analyses. First, we compared the latencies between trials 1 and 2 of the retraining day and found a significant reduction from trial 1 to 2 (Fig. 3C; trial 1: 33.5 ± 8.5 s; trial 2: 12.7 ± 3.7 s, mean ± s.e.m.; p < 0.04 in a two-tailed paired *t*-test). Although an increase in trial 3 was not expected, our results indicate that the rats learned the new platform position in the first trial. Second, we calculated the percentage of time that rats spent in each of the four quadrants of the water maze on the retraining day (Fig. 3D). In trials 1 and 2, we did not detect a significant bias in the percentage of time rats spent in each quadrant (trial 1: p > 0.5; trial 2: p > 0.08 in one-way ANOVA). On the other hand, rats showed significant biases in trials 3 (p < 0.004) and 4 (p < 0.0002), indicating that rats learned the new platform positions during the retraining day. It is important to note that, although rats unexpectedly showed longer latencies in trial 3 than in trial 2, the high percentage of time spent in the target quadrant containing the platform indicates that rats learned the platform position in the first two trials. A two-way repeated measures ANOVA (Trial × Group) did not detect a significant difference in the latencies to the platform on the retraining day between the two groups (Group: p > 0.5; Trial × Group: p > 0.2). The total swimming distances were also not different between the groups (reference memory group: 217.5 ± 19.1 m; working memory group: 270.6 ± 19.9 m in mean ± s.e.m.; p > 0.05 in a two-tailed *t*-test). Swimming speed averaged over the 4 retraining trials did not differ between the groups (reference memory group: 27.1 ± 1.6 cm/s; working memory group: 25.4 ± 1.1 cm/s in mean ± s.e.m.; p > 0.05 in a two-tailed *t*-test).

### Effect on the survival of new neurons

**Figure 4:**
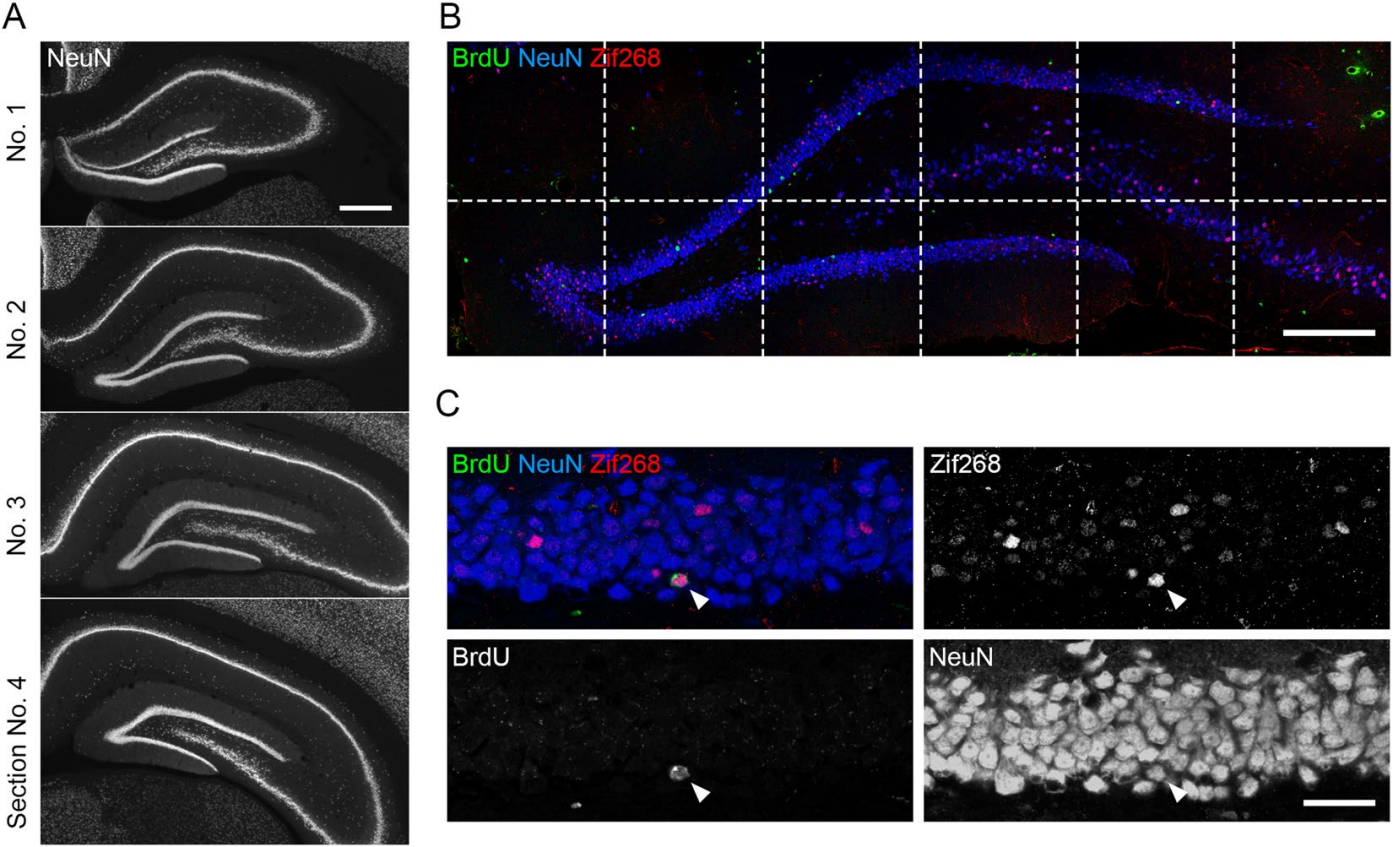
Immunofluorescence detection of BrdU-, NeuN-, and Zif268-positive cells. (**A**) Representative epifluorescence images from four sections used for the analyses. The sections were immunostained for NeuN. One in every 12 of the 40-µm-thick sections was selected, and the selected sections were designated #1–4. Scale bar: 500 µm. (**B**) An example of confocal image tiles, consisting of 12 images (512 × 512 pixels each) covering the whole granule cell layer. Blue: NeuN, red: Zif268, green: BrdU. Scale bar: 200 µm. (**C**) A confocal image showing BrdU/NeuN/Zif268-triple-positive cells (arrowheads). Scale bar: 30 µm.

**Figure 5:**
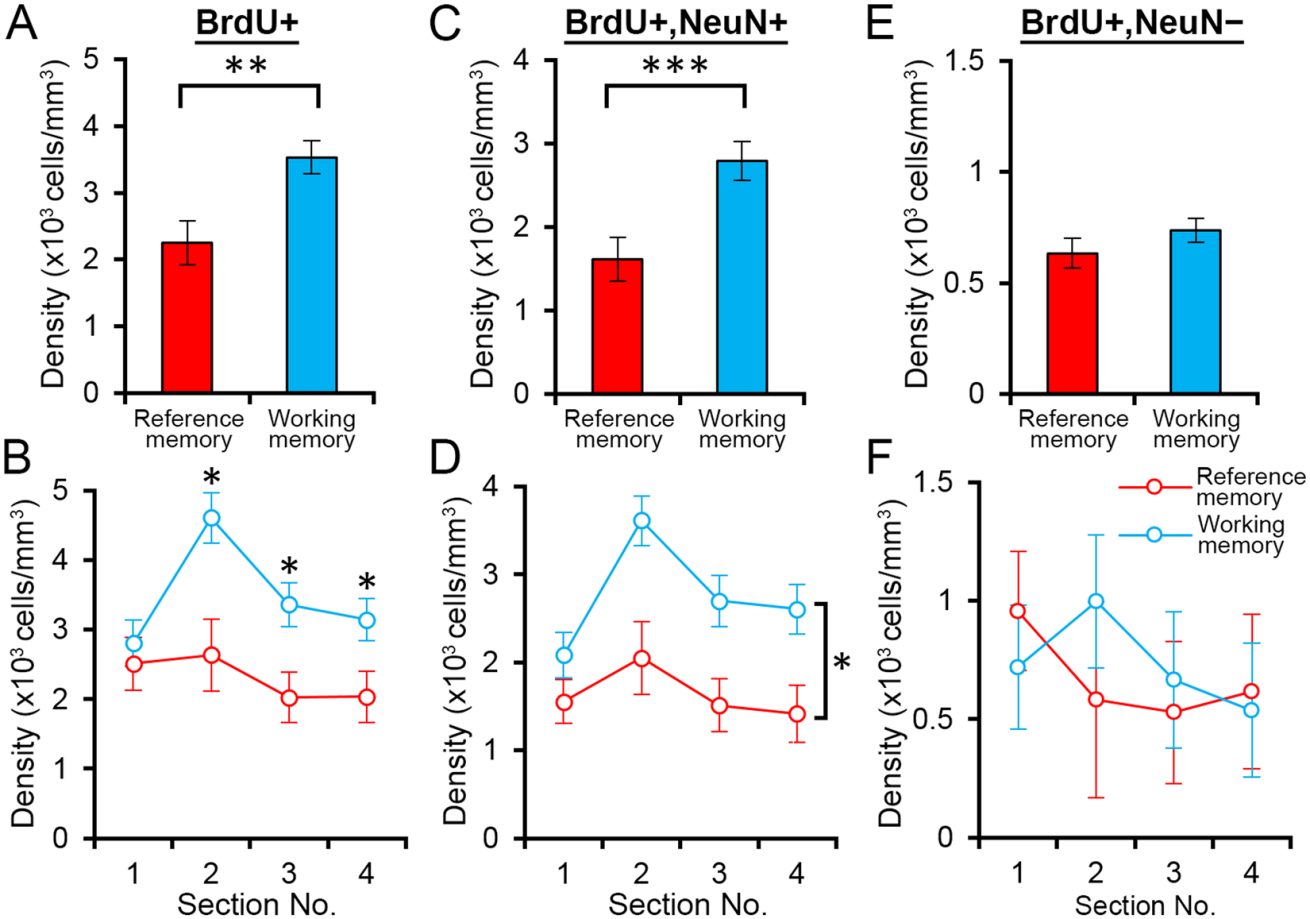
Density of BrdU-positive cells. (**A**,**C**,**E**) Overall densities of BrdU-positive (**A**); BrdU/NeuN-double-positive (**C**); and BrdU-positive, NeuN-negative (**E**) cells from all sections. **p < 0.01, ***p < 0.005 in a two-tailed *t*-test. (**B,D,F**) Mean densities in individual sections of BrdU-positive (**B**); BrdU/NeuN-double-positive (**D**); and BrdU-positive, NeuN-negative (**F**) cells. * in B: p < 0.05 in post hoc two-tailed *t*-test. * in D: p < 0.05 in Group effect, repeated measures ANOVA. All data are shown as the mean ± s.e.m.

Ninety minutes after the last trial on the retraining day, rats underwent perfusion fixation to prepare brain sections. The sections were sorted into anteroposterior sequences, and four sections (Fig. 4A; section No. 1, 2, 3, 4; 480 µm apart) were selected from each rat (reference memory group: n = 7 rats; working memory group: n = 8 rats) for confocal imaging (Fig. 4B,C). To examine the effects of the two tasks on the number of newly generated cells, we quantified the density of BrdU-positive cells. We found a significant difference between the groups (Fig. 5A; reference memory group: 2.25 ± 0.32 × 10^3^ cells/mm^3^, working memory group: 3.58 ± 0.25 × 10^3^ cells/mm^3^, in mean ± s.e.m.; p < 0.005, two-tailed *t*-test). To investigate the density in each section (section #1–4), we performed a two-way repeated measures ANOVA (Section × Group; Fig. 5B); we found significant effects of Section (p < 0.001) and Group (p < 0.02), and a significant Section × Group interaction (p < 0.03). Post hoc *t*-tests indicated significant between-group differences in sections 2, 3, and 4 (p < 0.04 for all).

To examine the effects of the tasks on the number of new neurons, we quantified the density of BrdU/NeuN-double-positive cells; we found a significant difference in the density of these double-positive cells between the groups (Fig. 5C; reference memory group: 1.62 ± 0.26 × 10^3^ cells/mm^3^; working memory group: 2.79 ± 0.23 × 10^3^ cells/mm^3^, mean ± s.e.m.; p < 0.005, two-tailed *t*-test). We performed a two-way repeated measures ANOVA (Section × Group; Fig. 5D) and found significant effects of Section (p < 0.0001) and Group (p < 0.01) but not a significant Section × Group interaction (p > 0.05). The differences between the groups indicate that training in the working memory task increased the survival of new neurons compared with training in the reference memory task. We also evaluated the effects on BrdU-positive, NeuN-negative cells and did not find a significant difference in their density between the groups (Fig. 5E, p > 0.05, two-tailed *t*-test). Two-way repeated measures ANOVA (Section × Group; Fig. 5F) did not detect a significant effect of Section or Group or a significant Section × Group interaction (p > 0.05). Therefore, this effect of increased survival is specific to new neurons.

**Figure 6:**
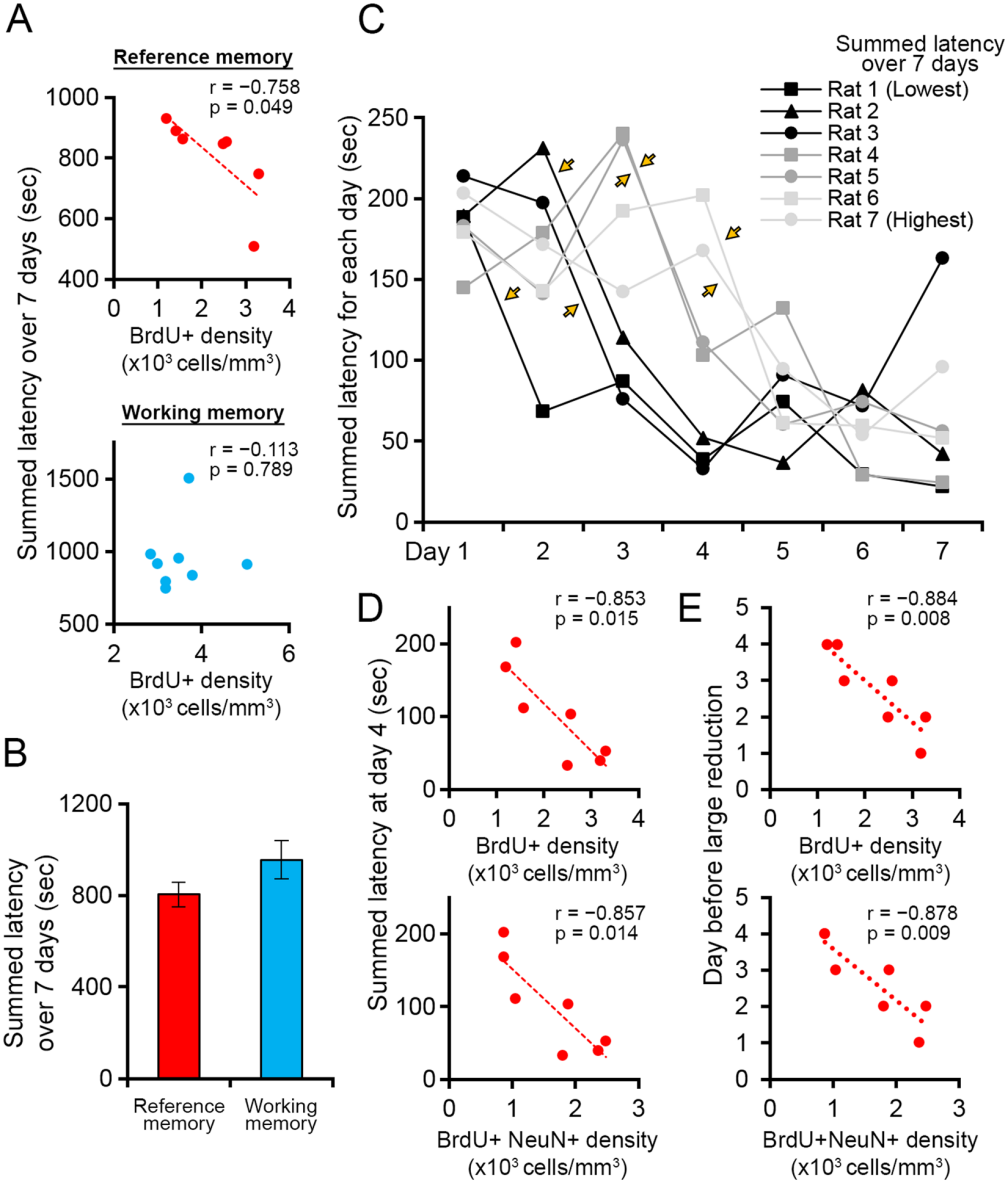
Correlation of reference memory task performance with the densities of BrdU-positive and BrdU/NeuN-double-positive cells. (**A**) Relationship between BrdU-positive cell density and summed latency over the 7-day training period. Top: reference memory group; bottom: working memory group. Each dot represents an individual rat. r: Pearson’s correlation coefficient; p: p-value for correlation (same for **D** and **E**). (**B**) Summed latency over the 7-day training period. Data are shown as the mean ± s.e.m. (**C**) Summed latency for all training days. Each line represents an individual rat. Orange arrows indicate the initial improvement of performance of individual rats. Rats are numbered/color coded according to the rank of their summed latency over the 7-day training period. (**D**) Correlation between summed latency on day 4 and the densities of BrdU-positive (top) and BrdU/NeuN-double-positive (bottom) cells. (**E**) Correlation between the day before a large reduction in latency occurred and the densities of BrdU-positive (top) and BrdU/NeuN-double-positive (bottom) cells.

Next, we examined the relationship between the survival effect and water maze performance. We first found that total latencies summed over the 7-day training period were significantly negatively correlated with BrdU-positive cell density in the reference memory group (Fig. 6A, Pearson’s correlation coefficient r = −0.758, p < 0.05), whereas no significant correlation was detected in the working memory group (p > 0.7). A possible interpretation of this correlation is that swimming duration is negatively correlated with BrdU-positive cell density. However, this interpretation that does not involve memory processes is unlikely because the working memory group, which swam for a comparable or slightly longer time (Fig. 6B), had a higher density of BrdU-positive cells than the reference memory group. Next, we examined in more detail how task performance and newly generated cell density are related. We examined changes in the task performance of individual rats over 7 days (Fig. 6C) and noticed that summed latency over 7 days seems to be closely related to two factors: latency on day 4 and how quickly spatial learning occurs. Regarding the latter, all the rats in the reference memory group showed a large, rapid reduction in latency between consecutive days (>70 s reduction, orange arrows in Fig. 6C), but the timing of this reduction was variable between rats. Some rats showed latency reductions early in the training period, resulting in shorter latency on day 4 (black data points). On the other hand, other rats showed this reduction later during the training period, leading to longer latency on day 4 (light gray data points). Both of these parameters are significantly negatively correlated with the densities of BrdU-positive cells and BrdU/NeuN-double-positive cells (Fig. 6D,E, p < 0.02 for all). Thus, the density of new neurons in the reference memory group is correlated with how quickly spatial learning occurs in the individual rats.

### Effect on the expression of Zif268

**Figure 7:**
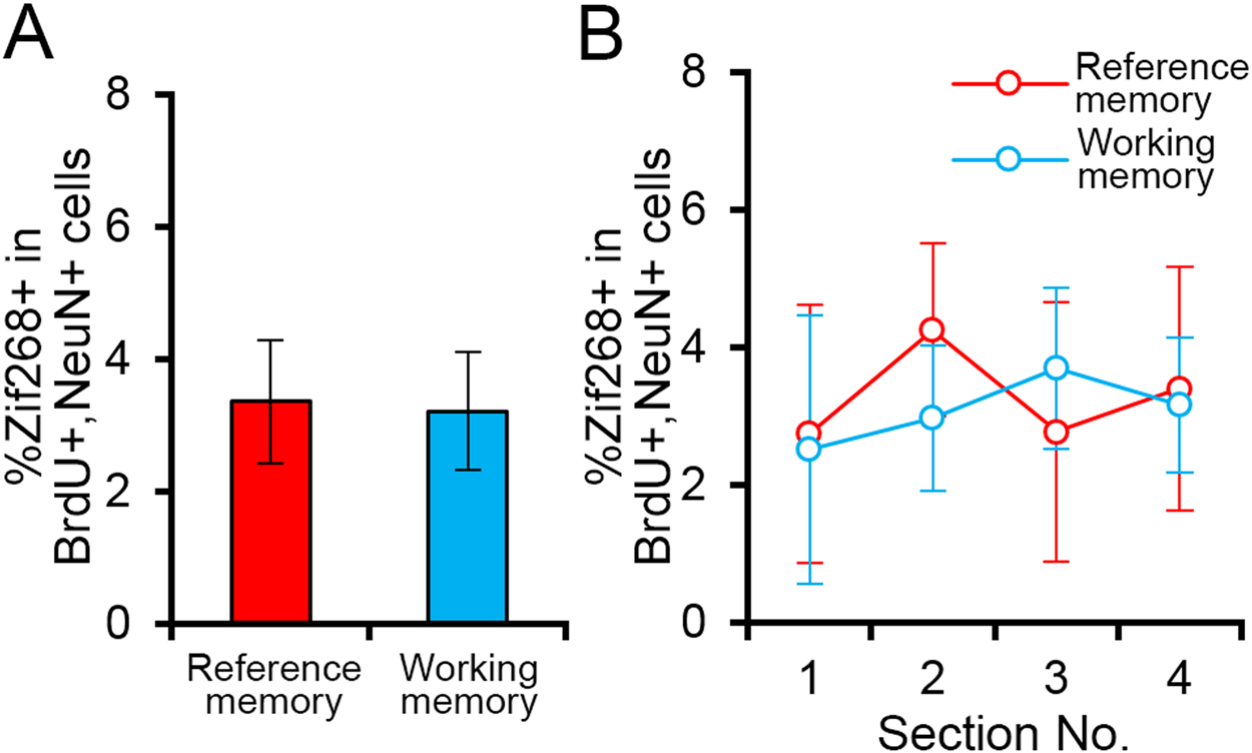
Zif268 expression in BrdU/NeuN-double-positive cells. (**A**) Mean overall percentages of BrdU/NeuN-double-positive cells expressing Zif268 from all sections. (**B**) Mean percentages of BrdU/NeuN-double-positive cells expressing Zif268 in the individual sections. All data are shown as the mean ± s.e.m.

Immediate early genes are a group of genes expressed rapidly and transiently in response to neuronal activation^24^, and their expression is frequently used as an indicator of neuronal activity. In this study, we used the expression of Zif268, which encodes a zinc finger transcription factor. We examined the degree of difference in the effects of the two tasks on Zif268 expression in new neurons after retraining; for this purpose, we quantified the percentage of BrdU/NeuN-double-positive cells that were positive for Zif268. We did not find a significant difference between groups (Fig. 7A; p > 0.05, two-tailed *t*-test). To examine the differences between groups in individual sections (#1–4), we performed a two-way repeated measures ANOVA (Section × Group); we did not detect a significant effect of Section or Group or a significant Section × Group interaction (p > 0.05 for all; Fig. 7B).

**Figure 8:**
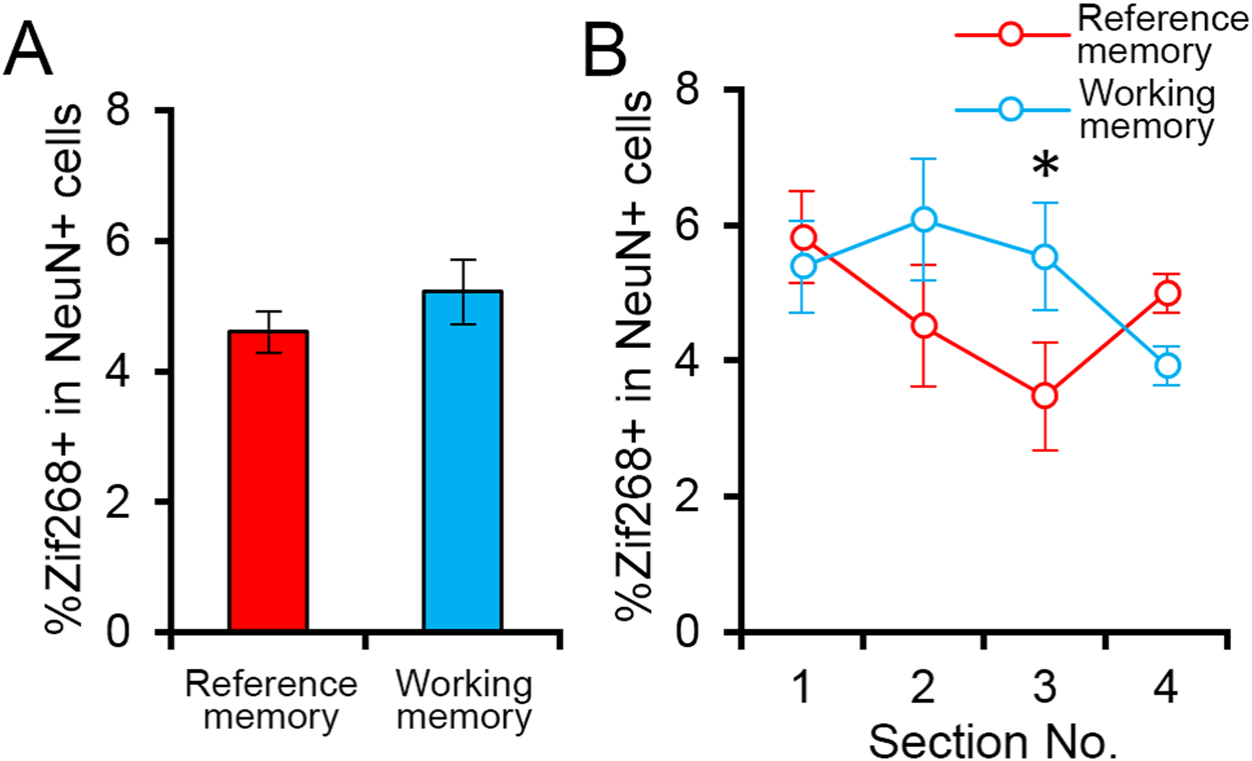
Zif268 expression in NeuN-positive cells. (**A**) Mean overall percentages of NeuN-positive cells expressing Zif268 from all sections. (**B**) Mean percentages of BrdU/NeuN-double-positive cells expressing Zif268 in the individual sections. *p < 0.05 in a post hoc two-tailed *t*-test. All data are shown as the mean ± s.e.m.

To compare the activation of new neurons with that of neurons in the granule cell layer, which consists mainly of mature granule cells, we also quantified the percentage of NeuN-positive cells that were positive for Zif268. We did not detect a significant overall difference between the groups (Fig. 8A; p > 0.05, two-tailed *t*-test). Two-way repeated measures ANOVA (Section × Group) detected a significant Section × Group interaction (Fig. 8B; p < 0.02). Post hoc *t*-tests detected significant differences between the groups in section #3 (p < 0.05) but not in the other sections (p > 0.05 for all).

## Discussion

Our study was motivated by the question of whether two memory subprocesses, i.e., acquisition and recall, differentially affect new neurons in the dentate gyrus. The ideal experiment would be to compare the effects of two tasks that involve either acquisition or recall exclusively. However, tasks purely involving a single memory subprocess are difficult to establish. Therefore, we decided to use the reference memory and working memory versions of the Morris water maze, since they involve different numbers of opportunities to acquire new information (i.e., more in the working memory task) and to recall previously acquired information (i.e., more in the reference memory task).

Our major finding is that the density of new neurons (BrdU/NeuN-double-positive cells) was significantly higher in the working memory group than in the reference memory group (Fig. 5). This result is aligned with findings from previous studies showing that animals’ experience during the first few weeks of neuronal birth has an impact on the survival or death of the new neurons^18,19,25–29^. Our result further extends this finding by suggesting that more opportunities to acquire new information may better support the survival of new neurons^21^. Multiple opportunities for memory acquisition heighten the cognitive resources required to successfully perform the task. This feature so called “cognitive demand” might determine this effect on new neurons, as proposed for the regulation of dendritic arbor formation^30^.

We also noted that performance in the reference memory task was correlated with the densities of BrdU-positive and BrdU/NeuN-double-positive cells (Fig. 6). Previous studies detected a similar correlation between the number of BrdU-positive cells and performance on memory tasks at similar time points after BrdU injections^31–33^. It is unclear how these correlations observed in our study and previous studies were generated. One possibility is that this correlation already existed before memory task training. A previous study suggested that the baseline level of adult neurogenesis may be correlated with memory task performance^34^. Therefore, rats with higher levels of adult neurogenesis may have shown faster learning in the reference memory task. Another possibility is that these correlations were caused by memory task training, as we observed that more new neurons survived in rats that showed faster learning. Although this correlation does not tell us how these parameters are mechanistically related, it is interesting that a similar correlation did not exist in the working memory group. Because of a ceiling effect, this correlation may exist only when animals have limited opportunities for memory acquisition and disappear if they undergo multiple opportunities for memory acquisition.

One previous study has examined the effects of tasks similar to the reference and working memory tasks used in our study^30^. The study found no difference in the survival of new neurons measured 14 days after neuronal birth, although differences were found in the morphology of the new neurons. Our results, taken together with this previous study, suggest that the differential effects on survival, which we observed at 6 weeks after neuronal birth and 4 weeks after training, may not have occurred immediately during the training period but after a delay. This possibility is consistent with previous findings that neuronal death, under basal conditions, continues to occur after 2 weeks of neuronal birth^35–38^. This period beyond 2 weeks corresponds to the period when the NMDA-receptor-mediated regulation of survival has been reported to occur^39^, suggesting that NMDA receptor activation may mediate the increased survival of new neurons induced by the working memory task.

We found a significant Section × Group interaction in the percentage of NeuN-positive cells that expressed Zif268 (Fig. 8B), which means that Zif268 expression patterns along the anteroposterior axis differ between the groups. Specifically, section 3 showed a significant group difference. These results indicate that the reference memory and working memory tasks activate the dentate gyrus differentially, supporting our experimental assumption that the two different water maze tasks use the dentate gyrus in dissimilar ways to meet distinct cognitive demands. By contrast, we did not find significant differences in the percentage of BrdU/NeuN-double-positive cells expressing Zif268 between the reference memory and working memory groups (Fig. 7C,D). Because the majority of NeuN-positive neurons are mature granule cells born in the developing brain, these results suggest that the two memory tasks recruit mature granule cells in the dorsal dentate gyrus differentially but activate new granule cells in a more similar way. This difference in activation supports the idea that adult-born neurons and mature granule cells have different roles in memory processes.

## Methods

### Subjects

Three-month-old female Long Evans rats were housed in cages on a 12-h:12-h light/dark cycle. The rats were intraperitoneally injected with BrdU (100 mg/kg body weight) every 12 h over 3 days (six injections in total) to label newborn neurons. The rats were handled by an experimenter during the week before training to minimize stress during the training. Starting from the seventh day after the last BrdU injection, the rats were trained with one of two versions of the Morris water maze tasks for a period of 7 days (reference memory group: n = 7 rats; working memory group: n = 8 rats, described below). Six weeks after the last BrdU injection, rats were retrained with the same water maze tasks. After the completion of the water maze retraining, the rats were wiped dry with a towel and kept in a dark room for 90 min. The rats were then deeply anesthetized with isoflurane followed by a lethal dose of Equithesin, after which they underwent perfusion fixation as previously described^40^. The timeline of the experiment is summarized in Fig. 1A. The experimental procedures with the rats were approved by the Norwegian Animal Research Authority and were performed in accordance with European convention and Norwegian regulation on Animal Experimentation.

### Morris water maze

The rats were divided into two groups: the reference memory group (n = 7 rats) and the working memory group (n = 8 rats). A circular pool 198 cm in diameter and 50 cm in depth was used for the water maze tasks. The pool was filled with water at 25 ± 1 °C, and the water was made opaque by dissolving non-toxic white paint in it (SchjerningFarver A/S, Denmark). A circular platform (11 cm in diameter) was hidden 1 cm below the water surface. For the reference memory group, the location of the hidden platform was kept constant over all training days, whereas the platform was moved into a new position every day for the working memory group (Fig. 1B). Each day, four trials were performed with fixed platform locations for both groups (Fig. 1C). Each trial started by placing a rat in one of four quadrants, facing the wall. The starting quadrants were pseudorandomly chosen, and trials lasted a maximum of 60 s followed by a 30-s rest on the platform. If a rat failed to find the platform within 60 s, an experimenter guided it to the platform location by placing one finger in the water in front of the rat. During a 10-s inter-trial interval, the rats were placed in their home cages. After four trials, the rats were warmed up under a heating lamp; however, this heating session was not performed on the final day in order to avoid any effects it might have on neuronal activation. Black cues in different shapes were placed on walls of the room. All trials were recorded with a video camera mounted over the pool, and the movement of the rats in all trials was tracked with a commercial online video-tracking system (DacqWM with Axonavideotracker, TRK-02, Axona Ltd, UK) and later analyzed using custom-written programs in MATLAB (MathWorks, USA).

### Immunohistochemistry

After perfusion fixation, 40-μm-thick coronal brain sections were prepared using a freezing microtome at −36 °C as previously described^40^. Eight sections taken every 480 μm, spanning the whole anteroposterior axis of the hippocampus, were immunostained for each animal. The sections were pretreated with 50% formamide at 65 °C for 2 h and then with 2N HCl at 37 °C for 30 min. This was followed by incubation with primary antibodies for 3 days at 4 °C, after which the sections were then incubated with secondary antibodies for 4 h incubation at room temperature. The primary antibodies used were rat anti-BrdU (1:400, AbD Serotec), rabbit anti-Zif268 (Egr-1) (1:400, Santa Cruz Biotechnology), and mouse anti-NeuN (1:400, Chemicon, Millipore). The secondary antibodies used were DyLight 488-conjugated donkey anti-rat IgG, DyLight 649-conjugated donkey anti-mouse IgG, and DyLight 549-conjugated donkey anti-rabbit IgG (1:250, Jackson ImmunoResearch Laboratories, Inc.). After immunostaining, all sections were mounted on Polysine slides with an antifade reagent.

### Microscopy and image analysis

Sections were sorted into anteroposterior sequences. Four middle sections (Fig. 4A; section #s 1, 2, 3, 4; 480 µm apart, 2.04–4.44 mm posterior to the bregma) were chosen from each rat for imaging (reference memory group: n = 7 rats; working memory group: n = 8 rats). Z-stacks of tiled images covering the whole granule cell layer (Fig. 4B,C) were acquired from the four sections by use of an LSM710 confocal microscope (Zeiss, Germany) equipped with 488-nm, 543-nm, and 633-nm laser lines, ZEN image-acquisition software and a 40× objective lens (NA 1.3). The z-stacks were analyzed by use of an image processing program, ImageJ (National Institutes of Health, USA). The area formed by densely packed NeuN-positive cells in the dentate gyrus was defined as the granule cell layer. The subgranular zone was defined as the area within the diameter of two cell bodies from the granule cell layer towards the hilus. For the purpose of counting BrdU/NeuN-double-positive cells (with or without Zif268 immunoreactivity), all cells in the granule cell layer and subgranular zone in the z-stacks were analyzed. For Figs 5A,C,E and 6A,B,D,E, the total number of cells from all four sections of each rat was divided by the total volume of the granule cell layer from all four sections of each rat to calculate a density value for each rat (reference memory group: n = 7 rats; working memory group: n = 8 rats). For Fig. 7A,the total number of Zif268/BrdU/NeuN-triple-positive cells from all four sections of each rat was divided by the total number of BrdU/NeuN-double-positive cells from the same four sections of each rat to calculate a percentage value for each rat (reference memory group: n = 7 rats; working memory group: n = 8 rats). For Fig. 5B,D,F, the total number of cells from each section of each rat was divided by the total volume of the granule cell layer from the same section to calculate a percentage value for each of the four sections of each rat (reference memory group: n = 7 rats; working memory group: n = 8 rats). For Fig. 7B, the total number of Zif268/BrdU/NeuN-triple-positive cells from each of four sections of each rat was divided by the total number of BrdU/NeuN-double-positive cells from the same section to calculate a percentage value for each of the four sections of each rat (reference memory group: n = 7 rats; working memory group: n = 8 rats). For the purpose of counting NeuN-positive cells (with or without Zif268 immunoreactivity), four areas per section (two areas each from the supra- and infragranular blades), containing ~100 cells from each section, were analyzed. For Fig. 8A, the total number of Zif268/NeuN-double-positive cells from all four areas of all four sections of each rat was divided by the total number of BrdU/NeuN-double-positive cells from the same areas to calculate a percentage value for each rat (reference memory group: n = 7 rats; working memory group: n = 8 rats). For Fig. 8B, the total number of Zif268/NeuN-double-positive cells from all four areas of each of four sections of each rat was divided by the total number of NeuN-positive cells from the same areas to calculate a percentage value for each of the four sections of each rat (reference memory group: n = 7 rats; working memory group: n = 8 rats).

### Data availability

The datasets generated during and/or analyzed during the current study are available from the corresponding author (A.T.) on reasonable request.
